# Regulation of Glutamate Signaling in the Sensorimotor Circuit by CASY-1A/Calsyntenin in *Caenorhabditis elegans*

**DOI:** 10.1534/genetics.118.300834

**Published:** 2018-02-23

**Authors:** Shruti Thapliyal, Shruthi Ravindranath, Kavita Babu

**Affiliations:** Department of Biological Sciences, Indian Institute of Science Education and Research (IISER), Manauli, Mohali 140306, Punjab, India

**Keywords:** *C. elegans*, CASY-1A, Glutamate, Sensory neurons, Neuromuscular junction

## Abstract

Locomotion is one of the most prominent behaviors in the nematode *Caenorhabditis elegans*. Neuronal circuits that ultimately produce coordinated dorso-ventral sinusoidal bends mediate this behavior. Synchronized locomotion requires an intricate balance between excitation and inhibition at the neuromuscular junctions (NMJ), the complex cellular and molecular mechanisms of which are not fully understood. Here, we describe the role of a cell adhesion molecule CASY-1, which functions to maintain this balance at the NMJ. In this study, we dissect out mechanisms by which the longer CASY-1A isoform could be affecting the excitatory cholinergic signaling at the NMJ by modulating the activity of sensory neurons. Mutants in *casy-1* appear to have hyperactive sensory neurons, resulting in accelerated locomotion and motor circuit activity. These sensory neurons mediate increased motor activity via enhanced glutamate release. Using genetic, pharmacological, and optogenetic manipulations, we establish that CASY-1A is required to monitor the activity of these neurons. Our study illustrates a novel neuromodulatory role of CASY-1-mediated signaling in regulating the excitation-inhibition balance of the motor circuit.

BEHAVIORAL output of an organism depends on the combined activity of multiple neural networks in which individual circuits are either activated or inhibited by the action of neurotransmitters or modified through neuropeptides. Locomotion is a very basic yet complex behavior in most organisms. An in-depth understanding of the *Caenorhabditis elegans* neural connectome provides an excellent model with which to understand the complex molecular and cellular mechanisms operating in locomotory circuits. Neural circuits that produce coordinated dorso-ventral sinusoidal bends allow for normal locomotion in *C. elegans*. Locomotory behavior is synchronized at multiple levels and involves the integration of diverse sensory cues that are processed by the interneurons and ultimately cause changes at the neuromuscular junctions (NMJ) (de Bono and Maricq 2005; Bargmann 2012). At the *C. elegans* NMJ, cholinergic motor neurons stimulate muscle contraction as well as activate GABAergic motor neurons that inhibit contraction of the contralateral muscles (White *et al.* 1986; Alfonso *et al.* 1993; McIntire *et al.* 1993a,b; Duerr *et al.* 2008). Despite an extensive understanding of the development and functioning of the ventral cord motor neurons, the mechanisms controlling motor coordination are still elusive. In mammals, disruption in this motor activity synchronization results in an excitation-inhibition imbalance that has been implicated in several neurological disorders like autism and epilepsy (Fetissov *et al.* 2003; Lerner *et al.* 2008; Wu *et al.* 2011). The *C. elegans* NMJ provides an excellent model to understand the genetic factors that coordinates this balance, thus providing deeper understanding of the pathogenesis of these disorders.

*C. elegans* CASY-1 is an ortholog of mammalian calsyntenin genes. Calsyntenins are Cadherin superfamily type-I transmembrane proteins characterized by the presence of two cadherin-like tandem repeats, an LG/LNS domain in the extracellular region, and an intracellular region that carries two kinesin light-chain binding domains (Hintsch *et al.* 2002; Araki *et al.* 2003, 2004). All these regions are conserved in the *C. elegans* calsyntenin ortholog, *casy-1*. Mammals have three calsyntenin genes (*clstn1*, *clstn2*, and *clstn3*) that are highly enriched in the nervous system (Hintsch *et al.* 2002). Similarly, *C. elegans* CASY-1 expression is also observed in most head neurons and some other tissues like intestine and gonadal sheath cells (Ikeda *et al.* 2008; Hoerndli *et al.* 2009). Polymorphisms of the human *clstn2* allele have been associated with increased episodic memory performance (Preuschhof *et al.* 2010). *C. elegans casy-1* has also been reported to be required for memory formation, thus suggesting conservation of functions (Ikeda *et al.* 2008; Hoerndli *et al.* 2009). Mammalian *clstn3* regulates excitatory and inhibitory synapse development in mice (Pettem *et al.* 2013; Um *et al.* 2014), while *clstn1* acts as a kinesin-1 adaptor that regulates trafficking and processing of amyloid precursor proteins (Konecna *et al.* 2006; Araki *et al.* 2007; Steuble *et al.* 2012; Vagnoni *et al.* 2012). Calsyntenins are also considered biomarkers for several age-related neurological disorders (Araki *et al.* 2003; Vagnoni *et al.* 2012; Uchida *et al.* 2013). Alterations in calsyntenin expression and function in neurological disorders make them potential candidates that can be explored further at the molecular and physiological levels for their potential roles in the pathogenesis of diseases.

In this study, we are proposing a novel neuromodulatory role of CASY-1-dependent signaling in the regulation of motor circuit dynamics and locomotion. We show that the CASY-1A isoform functions in the sensory neurons to inhibit the activity of command interneurons, thus negatively regulating motor circuit activity and locomotion. The CASY-1A isoform does this by controlling the release of glutamate from glutamatergic sensory neurons. Together, our results support a crucial role of CASY-1 in regulating locomotion dynamics by modulating the activity of the sensorimotor circuit.

## Materials and Methods

### *C. elegans* strain maintenance

Strains were maintained on nematode agar growth medium (NGM) seeded with OP50 *Escherichia coli* at 20° under standard conditions (Brenner 1974). The *C. elegans* N2 Bristol strain was used as the wild-type (WT) control. All experiments were carried out with young adult hermaphrodites at ∼23°. A complete list of strains utilized in this study is given in Supplemental Material, Tables S3 and S4 in File S1. The primers used for genotyping different mutant strains are tabulated in Table S5 in File S1. The N2 *C. elegans* and the OP50 *E. coli* were obtained from the *C. elegans* Genetics Center (University of Minnesota, Minneapolis, MN).

### Transgenic strains and constructs

Tables S1 and S2 in File S1 lists all the plasmids and constructs used in this study, Table S6 in File S1 lists the primers used to make the different transgenes. All the plasmids were generated using standard restriction digestion based cloning strategy and sequenced before use in the experiments. Transgenic strains were generated by previously described microinjection techniques to generate stable transgenic *C. elegans* lines carrying extrachromosomal DNA arrays using either p*myo-3*::mCherry, p*myo-2*::GFP, p*unc-122*::GFP, or p*vha-6*::mCherry as coinjection markers (Mello and Fire 1995).

### Aldicarb assay

The Aldicarb assays were performed as described previously (Mahoney *et al.* 2006b). NGM plates containing Aldicarb at a final concentration of 1 mM were used in this assay. For each assay, ∼20 young adult hermaphrodites were transferred onto Aldicarb plates and scored for paralysis every 15 min for >2.5 hr. Animals were considered paralyzed when they failed to show any body bends following prodding three times on the head.

For optogenetics-based Aldicarb assay, animals were exposed to a 1-min pulse of low-intensity blue light before, and after every 30 min during the Aldicarb assay. For this assay, Aldicarb plates were seeded with OP50 containing 0.8 mM all-trans retinal (ATR).

For histamine-induced silencing of command interneurons, NGM-HA plates were prepared. For this, 1 M histamine-dihydrochloride stock in water was added to NGM agar at ∼65° immediately before pouring plates (Pokala *et al.* 2014). Histamine-free control plates were poured from the same NGM batch. Histamine-containing plates were used for Aldicarb within 2 days after pouring.

All assays were performed with the experimenter blind to the genotypes. Each assay was performed at least three times or more as indicated at the base of each bar with >20 animals for each replicate. A graph was plotted with the average percentage of *C. elegans* that had paralyzed at 105 min.

The rate of Aldicarb-induced paralysis was found to vary in few of the experiments, although the Aldicarb concentration used in all the experiments was same. This variation could likely be due to different batches of Aldicarb used for these sets of experiments. However, the overall outcome of the result drawn from these assays was consistent for all the strains used in these assays.

### Locomotion assays

Well-fed synchronized animals were picked onto new 90 mm NGM plates seeded with a uniform lawn of OP50 *E. coli*. These plates were seeded 12 hr before the start of the assay and the bacteria were allowed to grow at 20°. The animals were allowed to equilibrate for 1 min after transfer to the assay plate. For all analysis, a 3-min video was made using a Zeiss Lumar V12 fluorescence Stereomicroscope. This video was then segmented into shorter videos of 20 sec using custom code written in MATLAB (MATLAB Release 2012b; The MathWorks, Natick, MA). The 20-sec-long video was then scored by eye for body bends. A body bend was defined as a change in the direction of propagation of the part of the animal corresponding to the posterior bulb of the pharynx along the *y*-axis, assuming the *C. elegans* was traveling along the *x*-axis (Tsalik and Hobert 2003).

### Imaging experiments

Animals were immobilized using 30 mg/ml 2,3-butanedione monoxamine on 2% agarose pads (Sieburth *et al.* 2005). Expression of transcriptional reporters iGluSnFR and SNB-1 was imaged using a Leica HC PL APO 63x/TCS SP8 confocal microscope (Leica Microsystems) with Multi-Ar (457, 488, and 515 nm), and He-Ne (543 and 633 nm) laser lines and HyD detectors. For iGluSnFR analysis, animals were imaged in the head region of the *C. elegans* for the AVA neuron cell body and axonal processes. For cell body measurements, images oriented sideways were included in the quantification analysis. For axonal fluorescence, worms positioned in dorsal up orientation were analyzed. For analysis of GABA motor neurons iGluSnFR, animals were imaged for the dorsal nerve cord in the posterior portion of the *C. elegans* halfway between the vulva and the tail. Quantitative imaging of GLR-1::GFP and NMR-1::GFP was performed using a Zeiss AxioImager microscope with a 63× 1.4 NA Plan APOCHROMAT objective equipped with a Zeiss AxioCam MRm CCD camera controlled by Axiovision software (Zeiss Micro-imaging). Animals were imaged for the ventral nerve cord in the posterior portion of the *C. elegans* halfway between the vulva and the tail. For fluorescent analysis, image stacks 10 µm optical slice (20 slices at 0.5 μm distance) were taken and maximum intensity projections were obtained using Image J software. The fluorescence intensity obtained from 15 to 25 animals was averaged and used to plot the graph.

### Statistical analysis

All statistical analysis was performed using GraphPad Prism V7. Experimental data are shown as mean ± SEM. Statistical comparisons were done using the Student’s *t*-test or one-way ANOVA with Bonferroni’s multiple comparison post-test. A level of *P* < 0.05 was considered significant.

### Data availability

Strains and plasmids are available upon request. Tables S1–S4 in File S1 contain all the plasmids and strains that have been used in this study.

## Results

### Mutants in *casy-1* have elevated Aldicarb sensitivity and locomotion rates

The *C. elegans* NMJ provides an excellent model with which to understand various aspects of synapse development and function. An intricate balance between the excitatory cholinergic and inhibitory GABAergic signaling maintains normal sinusoidal locomotion in *C. elegans*, and any imbalance in this signaling results in altered behavior (reviewed in Richmond 2005; Seifert *et al.* 2006).

**Figure 1:**
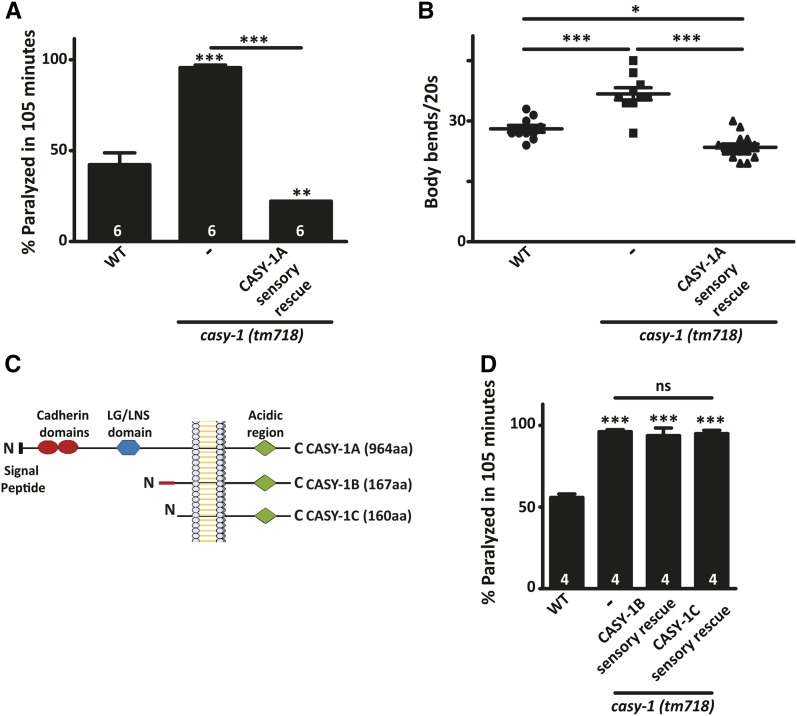
*casy-1* mutant animals have increased motor circuit activity. (A) Aldicarb-induced paralysis in *casy-1* mutants was compared to WT animals. *casy-1 (tm718)* mutants are hypersensitive to Aldicarb, which is completely rescued by expressing CASY-1A in sensory neurons using the *odr-4* promoter. Data are represented as mean ± SEM. The number of assays (∼20 *C. elegans*/assay) is indicated for each genotype. (B) *casy-1* mutants crawl faster than WT animals on agar surface seeded with OP50. Body bends/20 sec are shown for WT, *casy-1 (tm718)* and rescue lines expressing CASY-1A in sensory neurons. *n* = 10–15 animals. (C) A pictorial representation of the three CASY-1 isoforms—CASY-1A is a full-length protein with all the conserved domains—signal peptide (SP), two tandem cadherin domains, LG/LNS domain, transmembrane region, and cytosolic acidic region. CASY-1B and CASY-1C are truncated proteins lacking the extracellular LG/LNS domain as well as cadherin repeats. CASY-1B and CASY-1C differ in just seven amino acids at the N-terminal region, which are extra in CASY-1B. (D) Aldicarb-induced paralysis at 105 min, plotted for the indicated genotypes. Expressing CASY-1B or CASY-1C under the *odr-4* promoter in sensory neurons could not rescue the Aldicarb hypersensitivity of the *casy-1* mutants. Data are represented as mean ± SEM. The number of assays (∼20 *C. elegans*/assay) is indicated for each genotype. (* *P* < 0.01, ** *P* < 0.001, *** *P* < 0.0001 using one-way ANOVA and Bonferroni’s multiple comparison test).

Previously, an Aldicarb-based RNAi screen was performed to identify the role of cell adhesion molecules in NMJ functioning (Babu *et al.* 2011; and K. Babu and J. M. Kaplan, unpublished data). Aldicarb is a pharmacological inhibitor of the enzyme acetylcholine esterase that prevents the breakdown of excitatory neurotransmitter acetylcholine at the NMJ. WT *C. elegans* show a time-course-induced paralysis upon exposure to Aldicarb. It has been shown that mutations that increase synaptic transmission result in Aldicarb hypersensitivity, while mutations that decrease synaptic transmission result in Aldicarb resistance (Miller *et al.* 1996; Sieburth *et al.* 2005; Mahoney *et al.* 2006a; Vashlishan *et al.* 2008). RNAi against the *casy-1* gene resulted in Aldicarb hypersensitivity in this screen. To validate the results of the RNAi screening, the *casy-1* mutant allele, *tm718* was tested in the Aldicarb assay. *casy-1(tm718)* showed significant hypersensitivity to Aldicarb, suggesting neuromuscular signaling defects in these mutants (Figure 1A). Significant Aldicarb hypersensitivity in *casy-1* mutants also suggests an increased motor circuit activity (Fry *et al.* 2014). As locomotion directly reflects the coordination of motor circuit activity, the locomotory behavior of *casy-1* mutants on food was analyzed. To quantify the locomotory behavior, body bends of *C. elegans* on NGM agar seeded with bacteria were quantified. Although *casy-1* mutants maintain normal sinusoidal locomotion, they showed a significantly increased number of body bends in comparison to WT control animals (Figure 1B).

In *C. elegans*, CASY-1A expression is not seen in cholinergic or GABAergic ventral cord motor neurons (Thapliyal *et al.* 2018). However, it is strongly expressed in several sensory neurons and interneurons located in the anterior region of the animal (Figure S1 in File S1 and see Ikeda *et al.* 2008). Previous studies also support the absence of CASY-1A expression in ventral cord motor neurons. For instance, expression of CASY-1A in head neurons and not in cholinergic or GABAergic motor neurons has been previously described (Hoerndli *et al.* 2009). Some expression was seen in the ventral nerve cord, but that expression belongs to the axonal processes from the head neurons that traverse along the entire length of the *C. elegans* body. Moreover, the first study characterizing the *casy-1* gene in *C. elegans* also reported a similar expression pattern for *casy-1a* (Ikeda *et al.* 2008). Thus, based on two previously reported expression studies and our own results, we concluded that the CASY-1A isoform is unlikely to express in motor neurons *in vivo* in *C. elegans*. Recent publications have nicely highlighted how genes expressed in higher levels of locomotory circuits, *i.e.*, in sensory and interneurons, could affect synaptic signaling at the NMJ (Choi *et al.* 2013, 2015; Fry *et al.* 2014). Based on these previous publications, we hypothesized that the increased sensitivity to Aldicarb and the elevated locomotory rates in *casy-1* mutants could be due to increased signaling from the sensory neurons and/or interneurons. We were specifically interested in understanding the role of CASY-1A in sensory neurons, since previous reports have shown that learning deficits in *casy-1* mutants can be completely rescued by expressing CASY-1A in a single sensory neuron (Ikeda *et al.* 2008). Further, a salt-sensing neuron ASER has been reported to show increased synaptic transmission in *casy-1* mutants (Ohno *et al.* 2014). To test whether CASY-1A could function at the level of sensory neurons to regulate motor circuit activity, a transgenic line expressing CASY-1A in several sensory neurons using the *odr-4* promoter was obtained. Expressing CASY-1A in sensory neurons completely rescued the Aldicarb phenotype of *casy-1* mutants, in fact the animals became resistant to Aldicarb (Figure 1A). CASY-1A expression in sensory neurons also rescued the increased locomotory rate of *casy-1* mutants (Figure 1B). These results demonstrate that the CASY-1A isoform can regulate motor circuit activity from the sensory neurons. It also indicates that CASY-1A functions in the *C. elegans* nervous system to inhibit the motor circuit activity.

The *casy-1* gene locus in *C. elegans* encodes three isoforms; CASY-1A is a 984 residue full-length protein containing all the conserved domains of mammalian calsyntenins while CASY-1B and CASY-1C are truncated proteins encoding 167 and 160 residues, respectively, and lack most of the N-terminus of the calsyntenin gene (schematized in Figure 1C). To validate the exclusive role of CASY-1A isoform in regulating excitatory cholinergic transmission from sensory neurons and hence modulating the motor activity, transgenic lines expressing the shorter isoforms of CASY-1, CASY-1B, and CASY-1C in sensory neurons using the *odr-4* promoter were generated. These transgenic lines failed to rescue the Aldicarb hypersensitivity of *casy-1* mutants, thus confirming the role of the full-length CASY-1A isoform in this function (Figure 1D).

### CASY-1A affects glutamatergic signaling

CASY-1A expression in sensory neurons using the *odr-4* promoter rescued the Aldicarb hypersensitivity and increased locomotion rates of *casy-1* mutants. The *odr-4* promoter drives gene expression in 12 pairs of sensory neurons (ADF, ADL, ASG, ASH, ASI, ASJ, ASK, AWA, ASB, AWC, PHA, and PHB) (Dwyer *et al.* 1998). The neurotransmitter identity of these 12 neurons was next determined from previously published reports, and it appeared that ∼75% of these neurons are glutamatergic and release glutamate as the neurotransmitter (Gendrel *et al.* 2016). However, some sensory neurons also release acetylcholine as a neurotransmitter; hence, we cannot rule out a potential role for acetylcholine in the *casy-1* mutant phenotype. Nonetheless, this analysis raised the possibility that increased synaptic signaling and increased locomotion rates in *casy-1* mutants could be due to increased glutamatergic signaling at the sensory level. To test this hypothesis, several experiments were performed.

**Figure 2:**
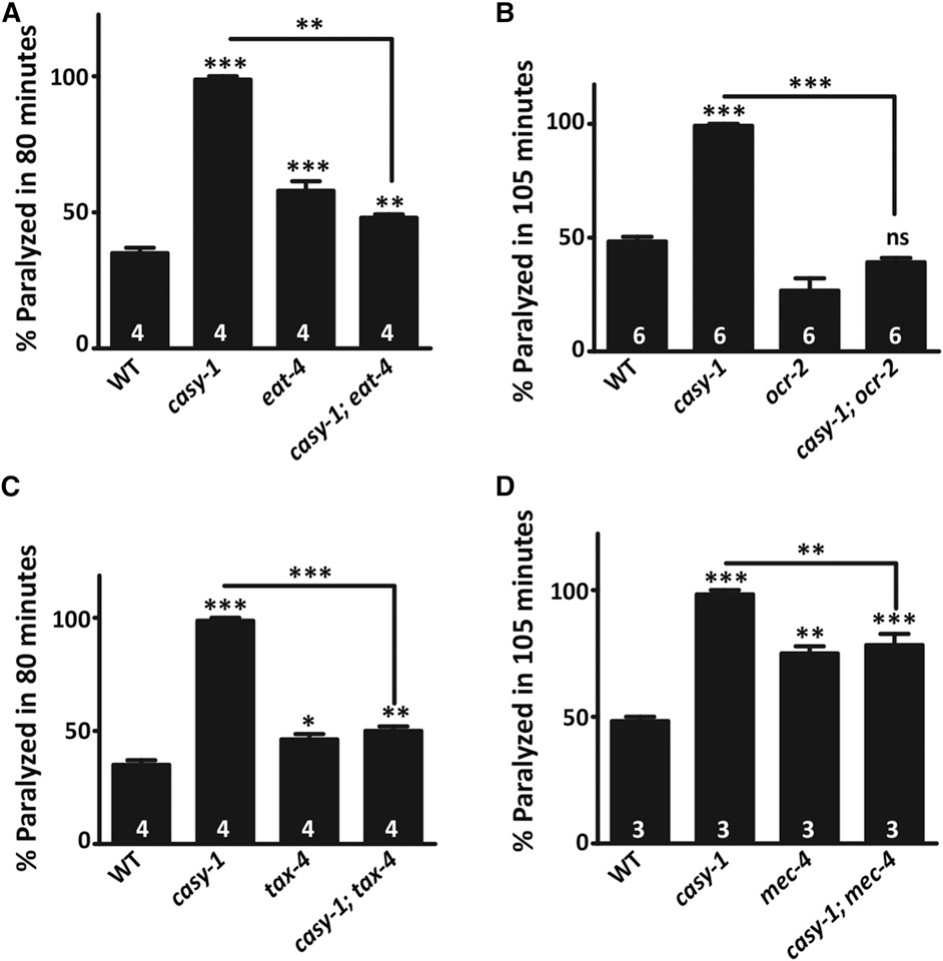
Motor circuit activity is enhanced due to increased sensory activity in *casy-1* mutants. (A–D) Aldicarb-induced paralysis at 80 or 105 min is shown for the indicated genotypes. The *casy-1* Aldicarb hypersensitivity was suppressed by mutations inactivating *eat-4/vglut*, *tax-4/cng* channels, *ocr-2/trpv* channels, and *mec-4/degenerin* amiloride-sensitive Na^+^ channels. Data are represented as mean ± SEM. The number of assays (∼20 *C. elegans*/assay) is indicated for each genotype. (* *P* < 0.01, ** *P* < 0.001, *** *P* < 0.0001 using one-way ANOVA and Bonferroni’s multiple comparison test).

To determine if increased glutamate signaling from sensory neurons mediates enhanced cholinergic transmission at the NMJ of *casy-1* mutants, *eat-4*/*vGLUT* (vesicular glutamate transporter) mutants, in which glutamatergic signaling is disrupted, were examined (Lee *et al.* 1999). The Aldicarb hypersensitivity in *casy-1* mutants was suppressed significantly by inactivating *eat-4/vGLUT*, indicating that the increased glutamate signaling facilitates sensory-evoked increase in motor circuit activity in *casy-1* mutants (Figure 2A). To address the reason for increased glutamatergic signaling, the Aldicarb sensitivity of *casy-1* mutants was analyzed by introducing mutations that inactivate ion channels required for sensory transduction; *ocr-2/trpv* (transient receptor potential channel), *tax-4/cng* (a cyclic nucleotide-gated channel), and *mec-4/degenerin* (amiloride-sensitive Na^+^ channel protein) (Driscoll and Chalfie 1991; Komatsu *et al.* 1996; Tobin *et al.* 2002). Mutations in these ion channels significantly diminished the Aldicarb hypersensitivity seen in *casy-1* mutants (Figure 2, B–D). Collectively, these results suggest that the Aldicarb hypersensitivity and increased locomotion in *casy-1* mutants is due to increased glutamatergic signaling triggered by heightened activity in the sensory circuit.

Recently, *casy-1* has been shown to interact with the neurexin-related protein *bam-2*, promoting fasciculation between sensory neurons required in mating circuits of the adult male (Kim and Emmons 2017), suggesting a role of *casy-1* in the proper development of sensory neuron networks. To determine if the altered glutamatergic signaling in *casy-1* mutants could be due to defects in the development of presynaptic glutamatergic synapses, a transgenic line was generated in which a *casy-1* expressing sensory neuron, ASH, could be visualized for the presynaptic SNB-1 puncta. SNB-1 is a *C. elegans* ortholog of the mammalian SV protein synaptobrevin. The SNB-1 mCherry fluorescence puncta were similar to WT control animals in *casy-1* mutants, suggesting presynaptic glutamatergic synapses are properly developed in ASH neurons (Figure S2 in File S1). However, this data cannot conclude normal development for all sensory neurons in *C. elegans casy-1* mutants.

### Glutamate release from sensory neurons is enhanced in *casy-1* mutants

**Figure 3:**
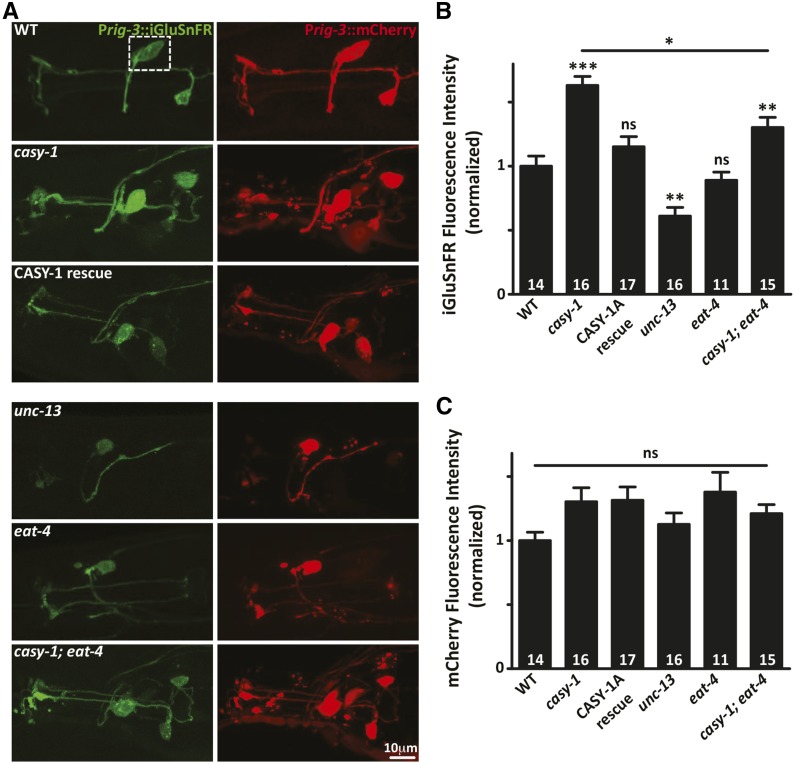
Glutamate release is enhanced in *casy-1* mutants. (A) Representative fluorescence micrographs of iGluSnFR expressed in AVA interneurons. Anterior is to the left in all images. iGluSnFR fluorescence intensity was significantly increased in *casy-1* mutants. Increased iGluSnFR fluorescence intensity was completely rescued by expressing CASY-1A in sensory neurons using *odr-4* promoter. iGluSnFR fluorescence intensity for *unc-13* mutants was also significantly reduced, thus serving as a positive control. The fluorescence intensity of vesicular glutamate transporter mutant *eat-4* was not different from WT animals; however, mutations in *eat-4* significantly suppressed the fluorescence intensity of *casy-1* mutants. (B) Quantification of iGluSnFR fluorescence intensity is shown for indicated genotypes. (C) Quantification of mCherry background reporter is shown for indicated genotypes. No significant change in mCherry fluorescence was observed thus negating changes due to array differences. Fluorescence intensity is normalized to WT values. The number of animals analyzed for each genotype is indicated at the base of the bar graph. Quantified data are displayed as mean ± SEM and were analyzed by one-way ANOVA and Bonferroni’s multiple comparison test (* *P* < 0.01, ** *P* < 0.001, *** *P* < 0.0001 using one-way ANOVA and Bonferroni’s multiple comparison test, “ns” indicates not significant in all figures).

One possible reason for increased glutamatergic signaling in *casy-1* mutants could be an increase in glutamate release from CASY-1-expressing sensory neurons. In order to investigate if *casy-1* mutants have increased glutamate release from the sensory neurons, a single wavelength glutamate sensor, iGluSnFR was used. iGluSnFR responds to the glutamate *in situ* and has been used for robust long-term imaging of glutamatergic transmission (Marvin *et al.* 2013). A transgenic line expressing iGluSnFR specifically in the AVA interneurons using the *rig-3* promoter was utilized for this experiment. To account for differences in array expression, another plasmid expressing mCherry under the same *rig-3* promoter was coinjected along with the iGluSnFR reporter. AVA is postsynaptic to 40 other neurons, many of which are glutamatergic (White *et al.* 1986). Glutamate input was monitored by measuring iGluSnFR fluorescence on the AVA cell body as well as along the axonal projections of AVA. iGluSnFR fluorescence increased significantly in the *casy-1* mutants, both in the cell body as well as in the axonal processes, indicating an increased release of glutamate from glutamatergic sensory neurons (Figure 3, A–C and Figure S3A in File S1). This increased iGluSnFR fluorescence was rescued completely by expressing CASY-1A specifically in sensory neurons using the *odr-4* promoter, thus validating the role of CASY-1A in regulating glutamate release from sensory neurons (Figure 3, A–C and Figure S3A in File S1). The iGluSnFR fluorescence intensity for synaptic vesicle release mutant *unc-13* was significantly reduced, serving as a positive control for this experiment (Figure 3, A–C). Interestingly, we found no significant change in the fluorescence intensity of *eat-4*/*vGLUT* (vesicular glutamate transporter) mutants, in which glutamatergic signaling is disrupted (Figure 3, A–C). However, removal of *eat-4* significantly suppressed the iGluSnFR intensity in the *casy-1* mutant background, thus supporting the results of our Aldicarb assays (Figure 3, A–C). One possible explanation for no change in fluorescence intensity in *eat-4* mutants could be the slow accumulated release of glutamate over time by the action of less characterized glutamate transporters *vglu-2* and *vglu-3* that could maintain basal levels of glutamatergic signaling in *eat-4* mutants. No significant change was observed in the fluorescence intensity of the mCherry marker among different genotypes, implying that the change in iGluSnFR fluorescence are due to changes in glutamate release from the presynaptic sensory neurons (Figure 3, A–C). Although nonsignificant, a slight variation in mCherry fluorescence among different genotypes could result from the fact that the transgenes used were extrachromosomal arrays.

The iGluSnFR fluorescence intensity was also monitored in the GABAergic motor neurons, which do not receive direct glutamatergic inputs and thus served as a negative control. A transgenic line expressing iGluSnFR reporter specifically in the GABA motor neurons using the *unc-30* promoter was generated. Several different transgenic lines were investigated as the reporter showed highly variable expression and very low fluorescence intensity. The iGluSnFR fluorescence on the dorsal cord of *casy-1* mutants was comparable to WT control animals, thus validating the specificity of this experiment (Figure S3B in File S1).

### Increased synaptic activity and glutamate release from sensory neurons is responsible for the *casy-1* locomotory and Aldicarb defects

**Figure 4:**
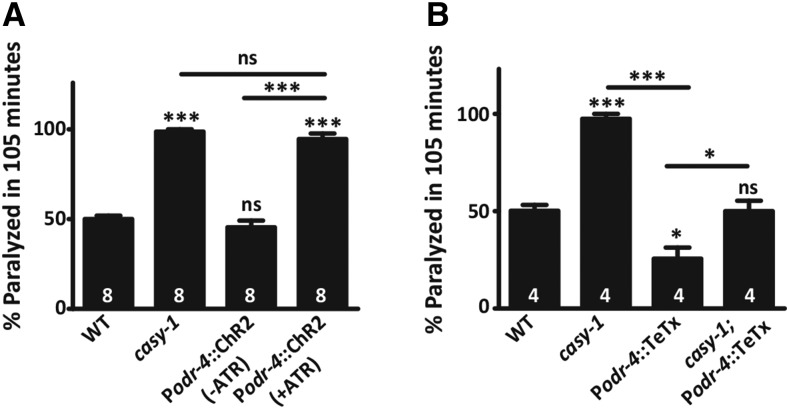
CASY-1A is required for the activity of sensory neurons, which promotes motor circuit activity. (A) Activation of sensory neurons using ChR2* (C128S) induces Aldicarb hypersensitivity in WT animals just like *casy-1* mutants. (B) Blocking vesicle release using sensory neuron-specific tetanus toxin (TeTx) induces Aldicarb resistance in WT animals. Blocking synaptic release from sensory neurons using TeTx, in the *casy-1* mutant background, causes the Aldicarb hypersensitivity of the *casy-1* mutants to reach WT levels. Promoter- intersectional, inverted Cre-Lox system is used to drive the expression of ChR2* (C128S) and TeTx in sensory neurons using *odr-4* promoter. Data are represented as mean ± SEM. The number of assays (∼20 *C. elegans*/assay) is indicated for each genotype. (* *P* < 0.01, ** *P* < 0.001, *** *P* < 0.0001 using one-way ANOVA and Bonferroni’s multiple comparison test, “ns” indicates not significant in all figures).

To gain insight into the role of CASY-1A in sensory neurons, an optogenetic approach was utilized wherein sensory neurons were photo activated using a slow inactivating variant of ChR2* (C128S) (Schultheis *et al.* 2011). The expression of ChR2* (C128S) was driven specifically in the sensory neurons using the *odr-4* promoter. To achieve this, a cell-specific, inverted Cre-Lox expression system was utilized, as previously reported (Flavell *et al.* 2013; Fry *et al.* 2014). Briefly, in this approach, one promoter drives the expression of floxed ChR2* and the GFP construct (using a pan-neuronal *rab-3* promoter) and a second neuron-specific promoter drives the expression of Cre (the recombinase). Thus, by promoter overlap, the expression of ChR2* will occur specifically in the neuron of interest (*odr-4* promoter driving expression in sensory neurons) (Flavell *et al.* 2013; Fry *et al.* 2014). This slow inactivating variant of ChR2* allowed us to monitor progressive paralysis in Aldicarb assays with intermittent blue light exposure (1-min pulse of blue light after every 30 min). Interestingly, in WT animals, photoactivation of sensory neurons results in Aldicarb hypersensitivity—a phenotype very similar to *casy-1* mutants. This experiment showed that increased activity of sensory neurons could result in Aldicarb hypersensitivity (Figure 4A and Figure S4A in File S1).

This was followed by a complementary experiment, where the possibility of vesicle release from the sensory neurons being responsible for increased Aldicarb hypersensitivity in *casy-1* mutants was addressed. The same promoter-intersectional, inverted Cre-Lox strategy was utilized to express tetanus toxin light chain (TeTx) specifically in the sensory neurons. TeTx blocks the release of vesicles containing both neurotransmitter and neuropeptides by cleaving the synaptic vesicle protein synaptobrevin (Schiavo *et al.* 1992; Sweeney *et al.* 1995; Fry *et al.* 2014). Blocking vesicle release from sensory neurons results in Aldicarb resistance in WT *C. elegans* (Figure 4B and Figure S4B in File S1). Next, rescue of *casy-1* hypersensitivity by blocking synaptic release from sensory neurons using P*odr-4*::TeTx was examined. Indeed, the *casy-1* mutant Aldicarb response was restored to WT levels upon blocking synaptic release in *casy-1* mutants (Figure 4B and Figure S4B in File S1). These results suggest that, in *casy-1* mutants, increased activity of sensory neurons trigger an increased release of glutamate and/or neuropeptide/s that facilitates an overall increase in the motor circuit activity. Collectively, these results suggest that in WT *C. elegans*, CASY-1A functions to regulate the activity of sensory neurons, which in turn allows for normal functioning of the *C. elegans* motor circuit.

### CASY-1A modulates motor circuit activity by inhibiting command interneuron signaling

Our results so far suggest that, in *casy-1* mutants, increased glutamatergic signaling from sensory neurons could affect motor neuron function. One possible way this could happen is through command interneurons that directly synapse onto the cholinergic motor neurons, and, hence, could allow for modulating the motor circuitry (White *et al.* 1976, 1986). In *C. elegans*, the neurotransmitter glutamate mediates synaptic transmission from sensory neurons to downstream interneurons and the command interneurons (Kaplan and Horvitz 1993; Brockie *et al.* 2001; Mellem *et al.* 2002).

**Figure 5:**
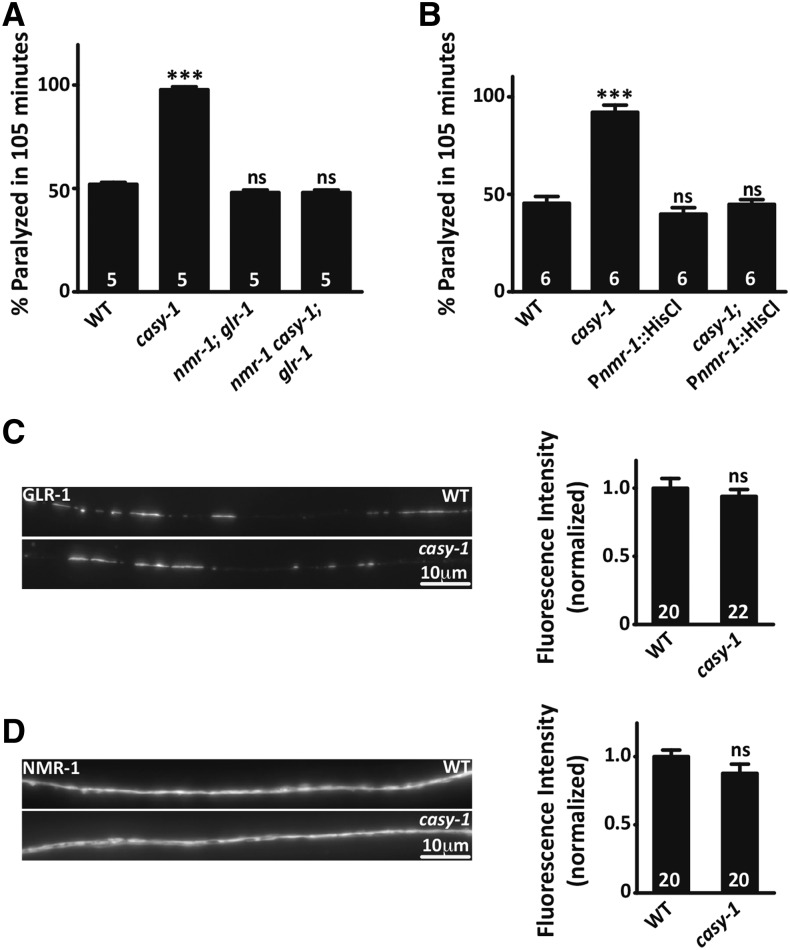
CASY-1A functions in sensory neurons to inhibit the command interneurons of the locomotion circuit to balance motor circuit activity. (A) Reduction of glutamate signaling in command interneurons using glutamate receptor (GluR) mutants suppresses the Aldicarb hypersensitivity of *casy-1* mutants. *nmr-1*; *glr-1* double mutant shows WT-Aldicarb response, and *nmr-1*; *glr-1* mutation in *casy-1* mutant background abolishes hypersensitivity in *casy-1* mutants. (B) Silencing command interneuron signaling using histamine-gated chloride channels under *nmr-1* promoter does not affect Aldicarb sensitivity in WT *C. elegans*, but significantly suppresses hypersensitivity in *casy-1* mutants. Data are represented as mean ± SEM. The number of assays (∼20 *C. elegans*/assay) is indicated for each genotype. (* *P* < 0.01, ** *P* < 0.001, *** *P* < 0.0001 using one-way ANOVA and Bonferroni’s multiple comparison test). (C and D) Representative fluorescent images showing GLR-1::GFP and NMR-1::GFP in posterior ventral nerve cord of WT and *casy-1* mutant animals. No significant difference was observed in fluorescence intensity compared to WT *C. elegans*. The number of animals analyzed for each genotype is indicated at the base of the bar graph. Quantified data are displayed as mean ± SEM and were analyzed by two-tailed Student’s *t*-test. “ns” indicates not significant in all figures.

To determine if the increased motor circuit activity in *casy-1* mutants is due to increased glutamate release from sensory neurons that in turn causes increased command interneuron signaling, several experiments were performed. The command interneurons AVA, AVB, AVD, AVE, and PVC are postsynaptic to sensory neurons as well as to several other interneurons (White *et al.* 1976, 1986). All these command interneurons express glutamate activated cation channels GLR-1/AMPA-type and NMR-1/ NMDA-type receptors that mediate excitatory neurotransmission (Hart *et al.* 1995; Maricq *et al.* 1995). If the hypersensitivity in *casy-1* mutants is due to increased glutamatergic signaling via command interneurons, then removing glutamate receptors should completely abolish the *casy-1* hypersensitivity. To discern this, *nmr-1*; *glr-1* double mutants and *nmr-1casy-1*; *glr-1* triple mutants were generated. The *nmr-1*; *glr-1* double mutants showed a WT response to Aldicarb. However, mutations inactivating *nmr-1* and *glr-1* in the *casy-1* mutant background completely abolished the Aldicarb hypersensitivity of *casy-1* mutants (Figure 5A).

Next, we silenced the command interneuron signaling using histamine-gated chloride channels, expressed specifically in command interneurons using the *nmr-1* promoter. Histamine-HisCl1 system has been well established to quickly and robustly silence *C. elegans* neurons and is often used as a useful complement to optogenetic approaches (Pokala *et al.* 2014). Silencing command interneurons in WT *C. elegans* results in a WT response to Aldicarb (Figure 5B and Figure S5 in File S1). This is not surprising as it has been shown previously that ablating command interneurons using cell-specific miniSOG (mini singlet oxygen generator)-based light-inducible cell ablation does not alter the Aldicarb hypersensitivity of the animals (Fry *et al.* 2014). However, silencing command interneuron signaling in *casy-1* mutants restored the Aldicarb response of *casy-1* mutants to WT levels, indicating that the command interneuron serves as a link downstream of CASY-1A in sensory neurons to modulate the motor circuit activity (Figure 5B and Figure S5 in File S1).

To further confirm that increased glutamatergic signaling is due to increased glutamate release from the sensory neurons and not due to increased expression of glutamate receptors on the postsynaptic interneurons, we imaged GLR-1::GFP and NMR-1::GFP in the *casy-1* mutant background. We found no significant change in the fluorescence intensity for both receptors, suggesting that the postsynaptic glutamate receptor levels are normal in the *casy-1* mutants (Figure 5, C and D).

Overall, our studies with *C. elegans casy-1* identify a novel regulator of excitation-inhibition balance at the NMJ that is required for efficient coordination of motor circuit activity and locomotion.

## Discussion

Locomotion is one of the most studied behavioral outputs in *C. elegans* and is mediated by a neuronal circuit that generates coordinated sinusoidal movement. The locomotor circuit in *C. elegans* works through a “cross-inhibition model” where the excitatory cholinergic motor neurons form dyadic synapses onto both the ipsilateral muscle as well as onto the inhibitory GABAergic motor neurons that then synapse onto the contralateral muscle (White *et al.* 1976, 1986). An important principle that maintains locomotion is a coordinated balance between the excitation (E) and inhibition (I) (Isaacson and Scanziani 2011; Stawicki *et al.* 2013). Despite reports on the involvement of E/I imbalance in the pathogenesis of several neurological disorders, genetic factors that control and modulate this balance have not all been identified. Previously, *acr-2(gf)* has been reported to alter this E/I balance resulting in spontaneous convulsions (Stawicki *et al.* 2013). More recently, the neuronal calcium sensor protein NCS-2 has been reported to regulate asynchronous release mediated E/I imbalance (Zhou *et al.* 2017). And the phosphorylation status of an endoplasmic-reticulum resident chaperone, RIC-3 has been shown to dictate the E/I balance at the muscle (Safdie *et al.* 2016). Our studies have identified CASY-1 as a regulator of both excitatory and inhibitory synaptic transmission, thus emerging as a novel controller of E/I balance.

We have recently shown that CASY-1 is required for regulating GABA release, and, hence, maintaining normal GABAergic neurotransmission at the *C. elegans* NMJ (Thapliyal *et al.* 2018). In this study, we report a possible mechanism for CASY-1A-mediated regulation of excitatory cholinergic transmission. Mutants in *casy-1* show enhanced locomotion on food, suggesting heightened motor circuit activity. Our results demonstrate that CASY-1A functions in multiple classes of sensory neurons to decrease the motor circuit activity. In this study, we propose one such mechanism that might explain the role of CASY-1A in this function. Mutants in *casy-1* have heightened sensory responsiveness, resulting in increased glutamate release, which in turn affects interneurons signaling and finally results in enhanced motor circuit activity.

**Figure 6:**
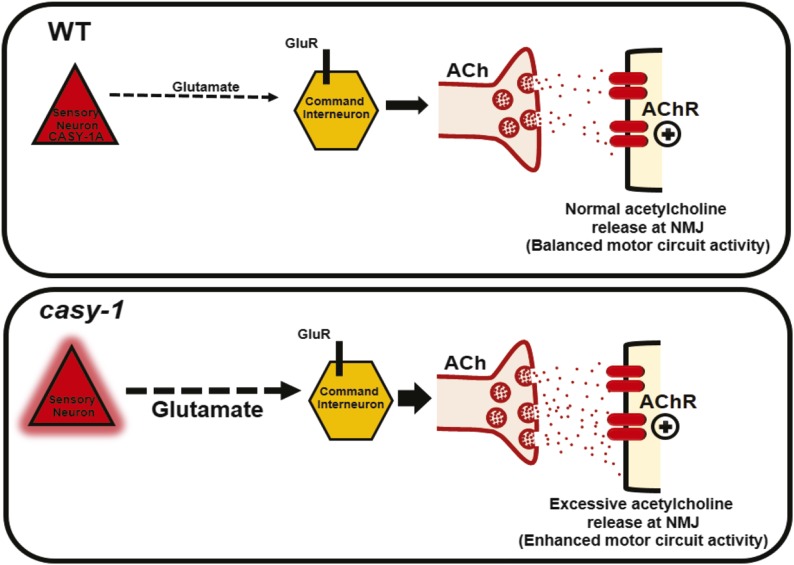
Proposed model for CASY-1A function in sensory neurons. Mutants in *casy-1* have an increased acetylcholine release at the NMJ. This study addresses the role of CASY-1A in maintaining the excitatory cholinergic transmission at the NMJ by employing multiple mechanisms of regulatory control from higher levels of locomotion circuit. In WT *C. elegans*, CASY-1A is expressed in sensory neurons to inhibit the motor circuit activity. In sensory neurons, it functions partly by regulating the release of neurotransmitter glutamate. Loss of *casy-1* results in enhanced glutamate release that exaggerates the motor circuit activity via increased signaling from command interneurons. Increased signaling from the command interneurons can in turn result in increased acetylcholine release at the NMJ, thus increasing the excitatory signals promoting enhanced motor circuit activity and Aldicarb hypersensitivity.

In WT *C. elegans*, CASY-1A is expressed in sensory neurons to inhibit the motor circuit activity. In sensory neurons, it functions partly by regulating the release of the neurotransmitter glutamate. Loss of *casy-1* results in enhanced glutamate release that could exaggerate the motor circuit activity through increased signaling from the command interneurons. Increased signaling from the command interneurons can in turn result in increased acetylcholine release at the NMJ, thus increasing the excitatory signals, leading to enhanced motor circuit activity and Aldicarb hypersensitivity (illustrated in Figure 6).

Our study proposes a model in which increased glutamatergic signaling from sensory neurons results in increased motor circuit activity, and, hence, Aldicarb hypersensitivity, in *casy-1* mutants. Interestingly, vesicular glutamate transporter mutant *eat-4* showed hypersensitivity in Aldicarb assays, although they have previously been shown to have a decreased glutamatergic signaling (Lee *et al.* 1999). Further, mutants for various ion channels required for sensory transduction in glutamatergic neurons like *tax-4* and *mec-4* are also expected to show decreased glutamatergic signaling. Previous reports have also presented data suggesting the hypersensitivity phenotype for *eat-4* and *tax-4* mutants on the Aldicarb assays (Choi *et al.* 2013, 2015). Further, mutations activating glutamatergic signaling have also been reported to show hypersensitivity in the Aldicarb assays (Fry *et al.* 2014). These studies report conflicting results for effect of glutamatergic activation on Aldicarb sensitivity. Future investigations uncovering the specific mechanisms for glutamatergic signaling regulated motor circuit activity might provide better insights into the role of mutants that show altered glutamatergic signaling.

Although this study focused mainly on the role of increased glutamatergic transmission from sensory neurons, *C. elegans* sensory neurons are also peptidergic and release neuropeptides along with classical neurotransmitters, and, hence, can act as diverse modulators of neural circuit function. Enhanced sensory activity in *casy-1* mutants might also play a role at the level of neuropeptide release from these neurons, thus adding another level of complexity to the regulation of motor circuit activity. Mammalian calsyntenins have not been addressed to interact with components of neuropeptidergic signaling. It will be interesting to investigate the functional role of *casy-1* in regulating neuropeptide-mediated behaviors.

Despite presenting a convincing mechanism for sensory-evoked enhancement for cholinergic transmission at NMJ, several questions in this study remains unanswered. For example, how exactly is CASY-1A involved in modulating the activity of sensory neurons? Our isoform-specific rescue experiments suggest that the CASY-1 C-terminal is dispensable for this function. The extracellular N-terminal region of CASY-1 harbors some conserved domains, such as cadherin and LG/LNS domains (Ikeda *et al.* 2008). Previously, the LG/LNS domain has been shown to be absolutely required for rescuing the learning deficits in *casy-1* mutants (Ikeda *et al.* 2008). It will be interesting to identify the role of the LG/LNS domain in regulating synaptic signaling at the NMJ. Also, identifying the interacting partners for *casy-1a* isoform could provide better mechanistic insights into this function. Another missing component of the study is how increased activity of sensory neurons could relate to enhanced acetylcholine release at the NMJ. Enhanced sensory activity could trigger several downstream signaling cascades that can function at multiple levels of the locomotion circuit. This study illustrates the role of glutamatergic signaling in sensory neurons conveying information to the motor circuit. However, some recent reports have described that several sensory neurons in *C. elegans* also utilize acetylcholine as a signaling neurotransmitter (Pereira *et al.* 2015), thus increasing the complexity of this investigation. Future studies concentrating on identifying the interacting partners of CASY-1A and understanding the role of other neurotransmitters might explain some of the results obtained in this study.

Mammalian calsyntenins have been reported to be required for the development and functioning of both excitatory glutamatergic and inhibitory GABAergic synapses (Pettem *et al.* 2013). Our study provides some useful insights into possible roles for mammalian calsyntenins at glutamatergic synapses. Substantial conservation of behavioral and genetic mechanisms between mammalian calsyntenins and *C. elegans* CASY-1A strengthens the likely existence of similar mechanisms in other systems.

## Supplementary Material

Supplemental material is available online at www.genetics.org/lookup/suppl/doi:10.1534/genetics.118.300834/-/DC1.
