# Supplementary material for: Regulation of Glutamate Signaling in the Sensorimotor Circuit by CASY-1A/Calsyntenin in Caenorhabditis elegans
Source: Genetics. 2018 Feb 23;208(4):1553–64. doi: 10.1534/genetics.118.300834 (PMC5887148; doi:10.1534/genetics.118.300834)
Supplement: Supplementary file 1 [file 1553FileS1.pdf]

Supplementary Material

Table S1: Plasmids obtained from other sources used in this study

Name	Description	Source
pKN22	P*rab-3*::*inverse Chr2*C128S*::SL2::GFP	K. Norman (FRY *et al.* 2014)
pKN24	P*rab-3*:: *inverse TeTx*::SL2::GFP	K. Norman (FRY *et al.* 2014)
pKN27	P*ver-3::nCre*	K. Norman (FRY *et al.* 2014)
pNP471	P*rig-3*::*HisCl1*::SL2::mCherry	C. Bargmann (POKALA *et al.* 2014)
pAG11	P*rig-3::iGluSnFR*::P*unc-122*::dsRed	C. Bargmann (MARVIN *et al.* 2013)
pCFJ90	P*myo-2*::mCherry	Addgene

Table S2: List of Plasmids and Arrays generated in this study

S.no.	Plasmid	Plasmid number	Array number
1	P*odr-4*::CASY-1B (injected into *casy-1*)	BAB1000	*IndEx1067*
2	P*odr-4*::CASY-1C (injected into *casy-1*)	BAB1001	*IndEx1068*
3	P*odr-4*:: nCRE (injected into N2) alongwith pKN22	BAB387	*IndEx1051*
4	P*odr-4*:: nCRE (injected into N2) alongwith pKN24	BAB387	*IndEx1052*
5	P*odr-4*:: nCRE (injected into *casy-1*) alongwith pKN24	BAB387	*indEx1053*
6	P*nmr-1::*HisCl (injected into N2)	BAB395	*indEx1043*
7	P*nmr-1::*HisCl (injected into *casy-1*)	BAB395	*indEx1044*
8	P*rig-3*::mCherry (injected into N2) alongwith pAG11	BAB1012	*indEx1059*
9	P*unc-30*::iGluSnFR (injected into N2)	BAB1003	*indEx1063*
10	P*nhr-79*::SNB-1::mCherry	BAB1009	*indEx1065*

Table S3: List of Integrated lines used in this study

S.no.	Plasmid	Integrated Line number	Strain number	Source
1	P*casy-1a*::GFP	*sIs10330*	BC11525	CGC

Table S4: List of strains generated in this study

S.no.	Genotype	Strain number	Source and reference

1	*casy-1(tm718)*		Y. Iino (IKEDA *et al.* 2008)
2	*casy-1 (tm718)* II; Ex[*odr-4p::casy-1, myo-3p*::venus]	JN485	Y. Iino (IKEDA *et al.* 2008)
3	*eat-4 (ky5)*	MT6308	CGC
4	*ocr-2 (ak47)*	CX4544	CGC
5	*tax-4 (p678)*	PR678	CGC
6	*mec-4 (u253)*	TU253	CGC
7	*eat-4 (ky5); casy-1(tm718)*	BAB1158	This study
8	*ocr-2 (ak47); casy-1(tm718)*	BAB1085	This study
9	*tax-4 (p678); casy-1(tm718)*	BAB1159	This study
10	*mec-4 (u253);casy-1(tm718)*	BAB1156	This study
11	*IndEx1051*	BAB1161	This study
12	*IndEx1052*	BAB1162	This study
13	*indEx1053*	BAB1163	This study
14	*glr-1 (n2461)*; *nmr-1(ak4)*	BAB1084	This study
15	*glr-1 (n2461)*; *nmr-1(ak4); casy-1(tm718)*	BAB1164	This study
16	*indEx1043*	BAB1143	This study
17	*indEx1044*	BAB1144	This study
18	*nuIs24* (P*glr-1*::GLR-1::GFP)		J. Kaplan (RONGO *et al.* 1998)
19	P*nmr-1*::NMR-1::GFP	VM484	CGC
20	BAB504; *casy-1(tm718)*	BAB168	This study
21	VM484; *casy-1(tm718)*	BAB170	This study
22	*IndEx1067*	BAB1195	This study
23	*IndEx1068*	BAB1196	This study
24	*indEx1059*	BAB1187	This study
25	*indEx1059; casy-1(tm718)*	BAB1188	This study
26	*indEx1059; eat-4 (ky5)*	BAB1189	This study
27	*indEx1059; unc-13 (s69)*	BAB1190	This study
28	*indEx1063*	BAB1191	This study
29	*indEx1063; casy-1(tm718)*	BAB1192	This study
30	*indEx1065*	BAB1193	This study
31	*indEx1059; casy-1(tm718)*	BAB1194	This study

Table S5: Primers utilized for genotyping

**Mutant genotyped**	**Primer number**	**Primer sequence**	**Mutation**
casy-1 (tm718)	ST150<br>ST151<br>ST152	gacgggtgatggaatgaaag<br>tcaaagcttctcctcccaga<br>cagcacgctcctacaacaag	Deletion

eat-4 (ky5)	ST509<br>ST510<br>ST511	gacgggtgggacccagcg<br>ggtgcagcacccccacagc<br>ctgagtgactaagtacccg	Deletion
tax-4 (p678)	ST512<br>ST513<br>ST514	ccagcggccaccggtgggc<br>ccagcggccaccggtgggt<br>tcacaaattttcatatgtggg	Substitution
ocr-2 (ak47)	ST515<br>ST516<br>ST517	cactagcagcatttaactgg<br>cgttcgccaaattctgcacc<br>gcttcatcaacagcttaccc	Deletion
mec-4 (u253)	ST635<br>ST636<br>ST637	cctctctgattgacattcttcc<br>cctggctcaaaaaatgctcc<br>catctgctcacggaattccc	Deletion
glr-1	AB037<br>AB038<br>AB039	acctttcggctccgacttg<br>acctttcggctccgactta<br>attgaaatgaccataccacc	Substitution
nmr-1(ak4)	ST366<br>ST367<br>ST368	gatgtgaggtgtccatgggc<br>gaagagtgcagtccagaggg<br>attcccttcaagcacctgctc	Deletion
unc-13 (s69)	ST385<br>ST386<br>ST387	cggaaatggcaaggcagg<br>actagatacatgtgCGCAG<br>ggagcagagactgctgacg	Deletion

Table S6: Primers utilized for cloning

FP denotes forward primer and RP, reverse primer

Gene/ Promoter cloned	Primer no.	Forward/ Reverse primer	Primer Sequence	Vector backbone
odr-4 promoter	ST638<br>ST639	FP<br>RP	ctctctgcatgcgaagccgcatcagaaactc<br>ctctctgctagcggattctgtaactggaattgc	pPD49.26 cloned with Cre recombinase
nmr-1 promoter	YD260<br>YD271	FP<br>RP	atagcctgcaggaagtggacactgagagagagag<br>agcaggtaccctgtaacaaaactaaagtttgtcgtgttc	pPD49.26/
rig-3 promoter	ST679	FP	ctctctgcatgcgaaatgcgacccatcatattgttcc	pPD49.26

	ST680	RP	ctctctggatcccaagtagtcgtccgattttcg	
nhr-79 promoter	ST646	FP	ctctctgcatgcggatagacttccagttgtg	pPD49.26
	ST647	RP	ctctctcccgggttttatgctaaaaatcgataaatcaagg	
unc-30 promoter	ST621	FP	ctctctctgcagcgccattcggagcacgctcttcagc	pPD49.26
	ST622	RP	ctctctggatccgccggagggcgcctcaatcccc	
CASY-1A gene	ST336	FP	ctctctggtaccatgcgaactgcgtactttatttttgtcg	pPD49.26
(for rescue)	ST162	RP	ctctctactagtggggaaggagtgaaaaggac	
CASY-1B gene	ST337	FP	ctctctggtaccatgttcgtgaacattctgg	pPD49.26
(for rescue)	ST162	RP	ctctctactagtggggaaggagtgaaaaggac	
CASY-1C gene	ST338	FP	ctctctggtaccatggacctcccgcgtccg	pPD49.26
(for rescue)	ST162	RP	ctctctactagtggggaaggagtgaaaaggac	
mCherry	ST404	FP	ctctctggatccatggtctcaaagggtgaagaag	pPD49.26
	ST506	RP	ctctctcccgggcttatacaattcatccatgcc	
HisCl	ST001	FP	caggaggacccttggctagcatgcaaagcccaacta<br>gcaa	pPD49.26<br>(amplified from<br>pNP471)
	ST002	RP	gatgagacagcggtacctcataggaacgttgtccaat	
SNB-1	ST677	FP	ctctctgctagcatggacgctcaaggagatgccgg	pPD49.26<br>(amplified from<br>genomic DNA)
	ST688	RP	ctctcttggccaatggacgctcaaggagatgccgg	

References

Fry, A. L., J. T. Laboy and K. R. Norman, 2014 VAV-1 acts in a single interneuron to inhibit motor circuit activity in Caenorhabditis elegans. Nat Commun 5**:** 5579.

Ikeda, D. D., Y. Duan, M. Matsuki, H. Kunitomo, H. Hutter *et al.*, 2008 CASY-1, an ortholog of calsyntenins/alcadeins, is essential for learning in Caenorhabditis elegans. Proc Natl Acad Sci U S A 105**:** 5260-5265.

Marvin, J. S., B. G. Borghuis, L. Tian, J. Cichon, M. T. Harnett *et al.*, 2013 An optimized fluorescent probe for visualizing glutamate neurotransmission. Nat Methods 10**:** 162-170.

Pokala, N., Q. Liu, A. Gordus and C. I. Bargmann, 2014 Inducible and titratable silencing of Caenorhabditis elegans neurons in vivo with histamine-gated chloride channels. Proc Natl Acad Sci U S A 111**:** 2770-2775.

Rongo, C., C. W. Whitfield, A. Rodal, S. K. Kim and J. M. Kaplan, 1998 LIN-10 is a shared component of the polarized protein localization pathways in neurons and epithelia. Cell 94**:** 751-759.

Fig. S1 (Supplement to Figure 1)

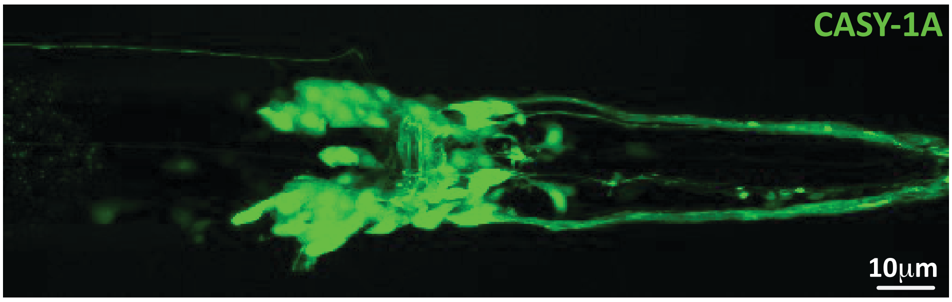

Figure S1. *casy-1a* expresses in head sensory and interneurons
Representative fluorescence micrograph showing P*casy-1a*::GFP transcriptional reporter expression in *C. elegans* head neurons.

Fig.S2 (Supplement to Figure 2)

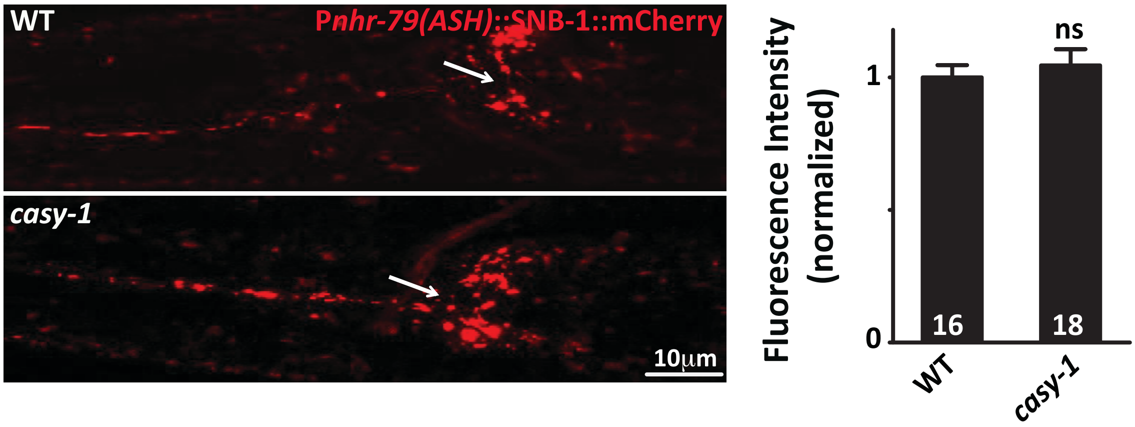

Figure S2. Presynaptic glutamatergic synapses are normal in *casy-1* mutants
(A) Representative fluorescence micrographs of SNB-1::mCherry expressed in ASH sensory neurons using *nhr-79* promoter. SNB-1::mCherry fluorescence was similar to WT control animals in *casy-1* mutants. Region quantified is indicated with an arrow. (B) Quantification of SNB-1::mCherry fluorescence intensity is shown for indicated genotypes. The quantification of fluorescent intensity is normalized to WT values. The number of animals analyzed for each genotype is indicated at the base of the bar graph. Quantified data are displayed as mean ± S.E.M. and were analyzed by two-tailed Student's t-test. "ns" indicates not significant.

Fig. S3 (Supplement to Figure 3)

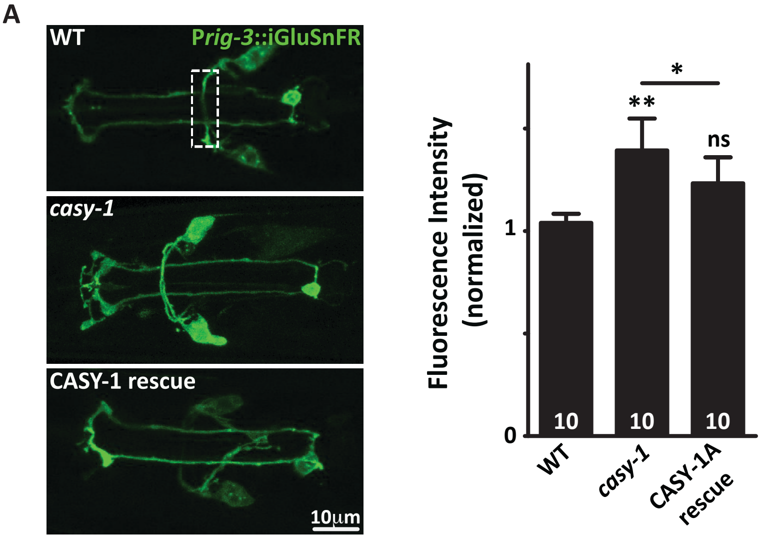

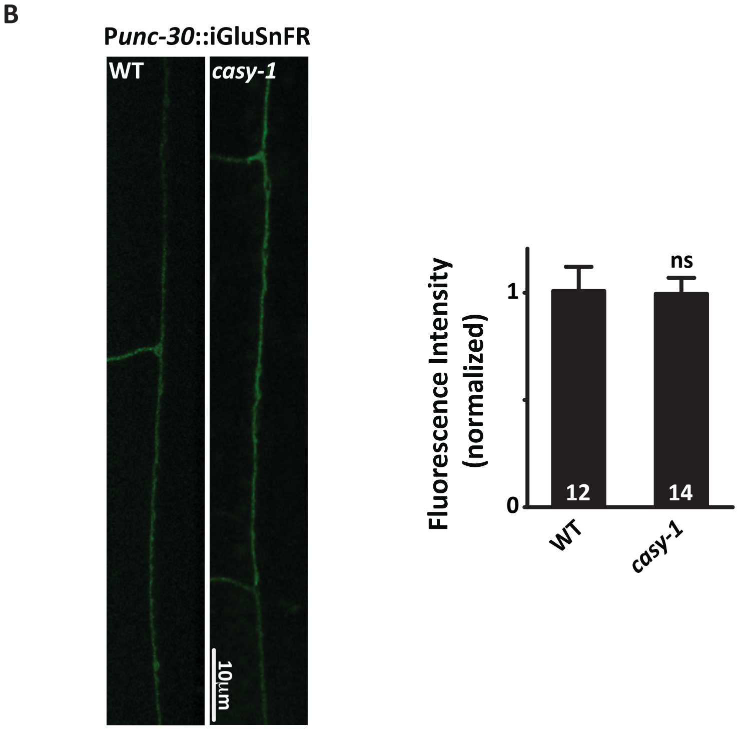

Figure S3. iGluSnFR axonal fluorescence is enhanced in *casy-1* mutants
(A) Representative fluorescence micrographs and quantitation of the fluorescence intensity of iGluSnFR expressed in the AVA interneurons. iGluSnFR axonal fluorescence was significantly increased in *casy-1* mutants. Further the increased iGluSnFR axonal fluorescence could be significantly rescued by expressing CASY-1A in the sensory neurons using the *odr-4* promoter. (B) Representative fluorescence micrograph and quantitation of the fluorescence intensity of iGluSnFR expressed in GABAergic motor neurons using the *unc-30* promoter. GABA motor neurons do not receive glutamatergic inputs and served as a negative control. iGluSnFR dorsal nerve cord fluorescence was similar to WT control animals in the *casy-1* mutants. The quantification of fluorescent intensity is normalized to WT values. The number of animals analyzed for each genotype is indicated at the base of the bar graph. Quantified data are displayed as mean ± S.E.M. and were analyzed by one-way ANOVA and Bonferroni's Multiple Comparison Test or two-tailed Student's t-test. (*p<0.01, **p<0.001 and "ns" indicates not significant).

Fig. S4 (Supplement to Figure 4)

A

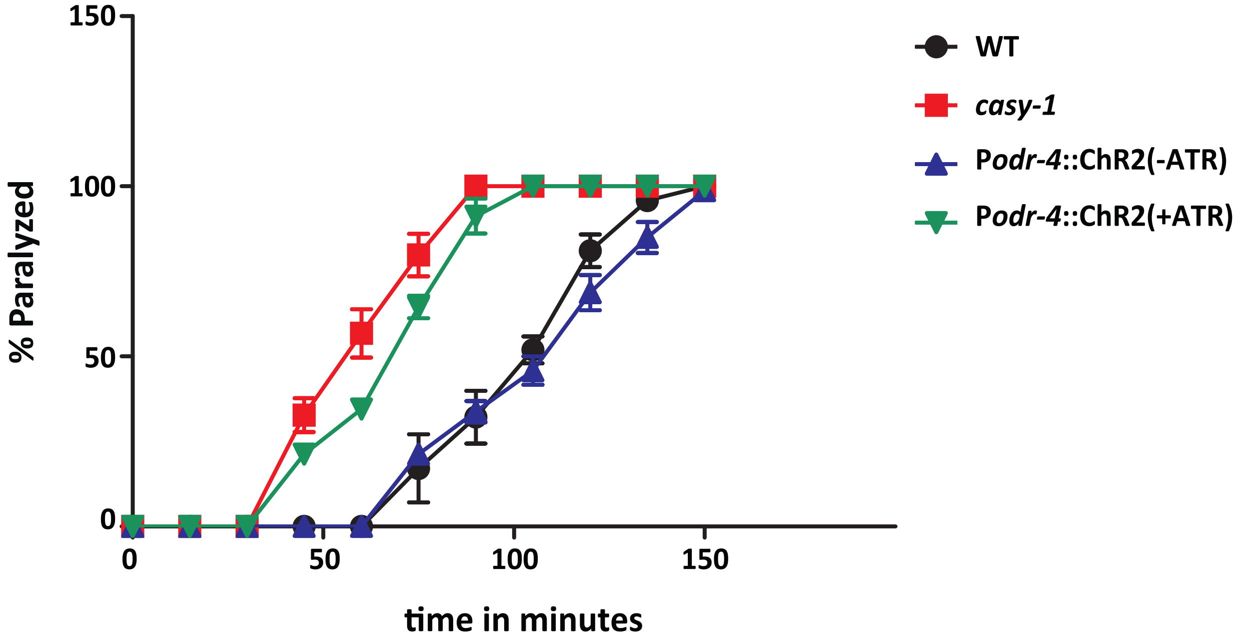

B

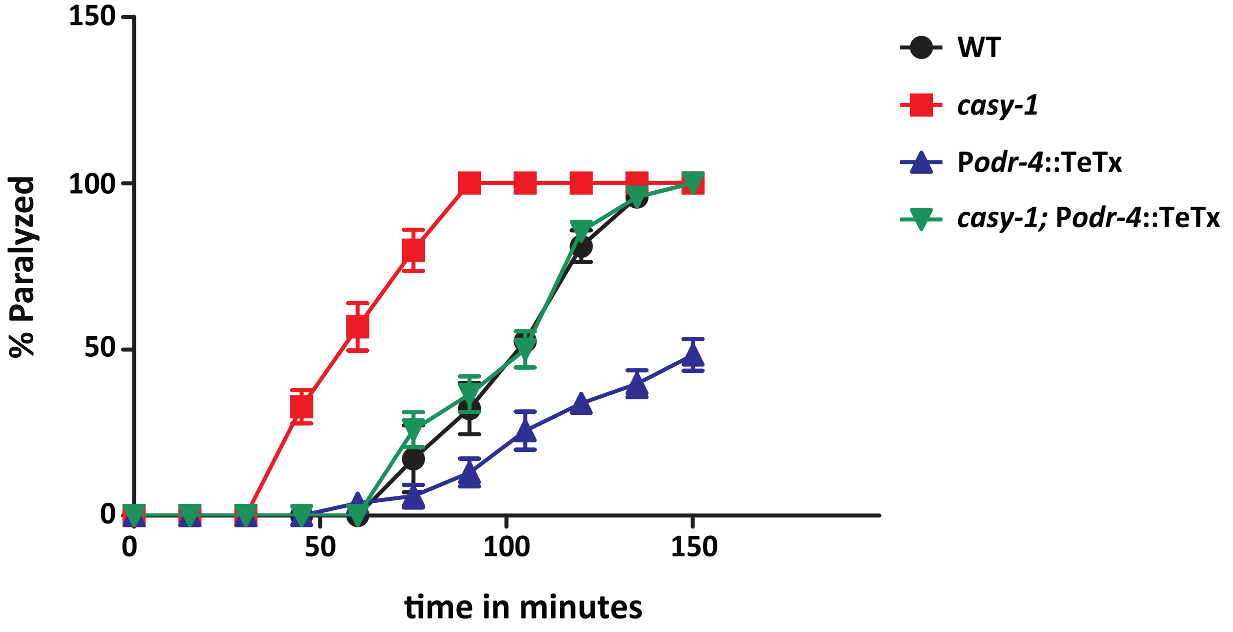

Figure S4. Time course of paralysis of animals treated with Aldicarb
Time-course experiments of 1mM Aldicarb- induced paralysis (upto 150 minutes) were plotted for the indicated genotypes. (A) Optogenetically activating sensory neurons using ChRX2* (C128S) under *odr-4* promoter results in hypersensitivity in WT animals. (B) Blocking vesicle release from sensory neurons using TeTx results in Aldicarb resistance in WT C. elegans, as well as suppresses the hypersensitivity seen in *casy-1* mutants to approximately WT levels.

Fig. S5 (Supplement to Figure 5)

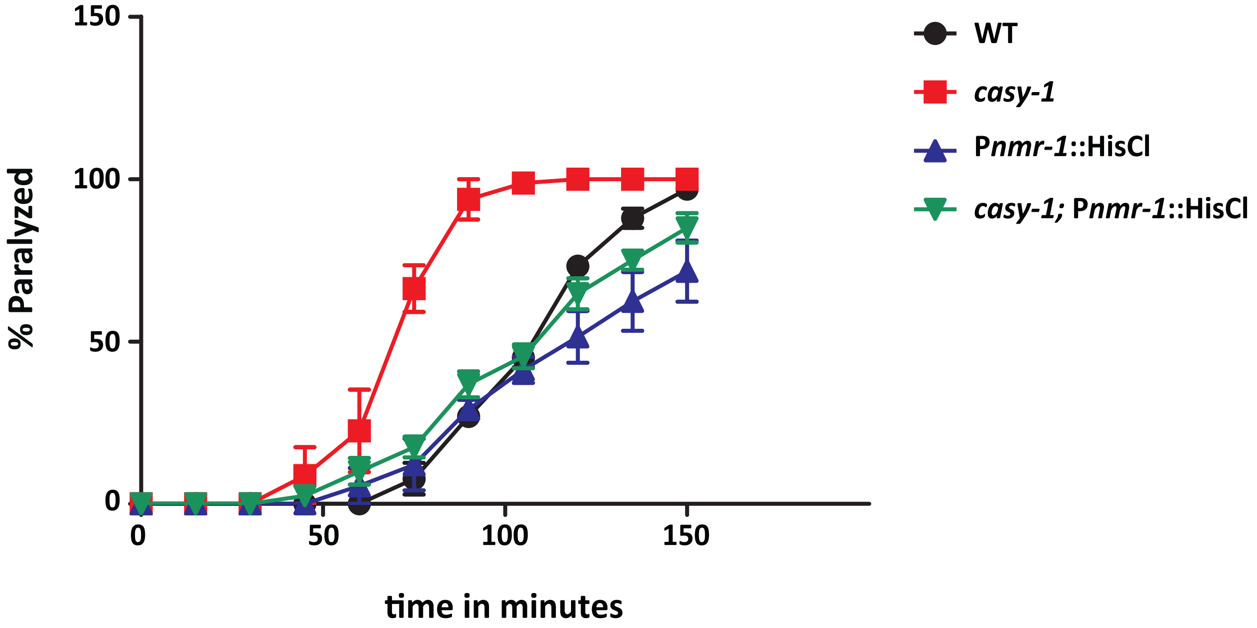

Figure S5. Time course of paralysis of *C. elegans* treated with Aldicarb
The time-course experiment of Aldicarb- induced paralysis (up to 150 minutes) plotted for the indicated genotypes. Silencing command interneurons using Histamine-gated chloride channels under *nmr-1* specific promoter does not affect the Aldicarb sensitivity of WT *C. elegans*, but significantly eliminates the Aldicarb hypersensitivity of *casy-1* mutants.
